# 异丙酯化衍生-气相色谱-质谱法测定烟草中29种有机酸

**DOI:** 10.3724/SP.J.1123.2025.01010

**Published:** 2025-11-08

**Authors:** Jie YU, Yang GAO, Da WU, Yuqi CAO, Dawei QI

**Affiliations:** 上海烟草集团有限责任公司技术中心，上海 201315; Technical Center，Shanghai Tobacco Group CO. ，LTD. ，Shanghai 201315，China

**Keywords:** 异丙酯化衍生, 气相色谱-质谱法, 烟草, 有机酸, isopropyl esterification, gas chromatography-mass spectrometry （GC-MS）, tobacco, organic acids

## Abstract

烟草中有机酸的组成与含量测定对烟草的品质评价具有重要意义，为实现烟草中挥发性、半挥发性和非挥发性有机酸的同时、准确、快速测定，本研究开发了一种异丙酯化衍生-气相色谱-质谱法同时测定烟草中29种有机酸的方法。研究系统优化了酯化衍生反应条件及实验参数，最终确定以10%硫酸异丙醇溶液（含0.1 g/mL对甲苯磺酸）为异丙酯化衍生试剂，衍生反应温度为70 ℃，反应时间为3 h，反应结束静置至室温后，加入10 mL去离子水，2 mL正己烷作为萃取溶剂，选用DB-FASTFAME色谱柱（30 m×0.25 mm×0.25 μm）进行程序升温分离，气相色谱-质谱法定量检测。在既定仪器参数及衍生化条件下进行方法验证，结果表明：29种有机酸在检测范围内线性良好，相关系数（*r* ^2^）均大于0.99，不同加标水平下的回收率为81.8%~118.7%，日内重复性为0.5%~8.1%，日间重复性为0.9%~8.2%，检出限（*S/N*=3）为0.01~49.02 μg/g，定量限（*S/N*=10）为0.03~163.41 μg/g。针对烟草中半挥发性及非挥发性有机酸，本方法与经典的甲酯化衍生法测定结果一致。将新开发的方法应用于烤烟、雪茄烟、白肋烟3种类型烟草样品的测定，均成功检出29种有机酸。该方法操作简便，检测通量高，实现了烟草中挥发性、半挥发性及非挥发性29种有机酸的同时、准确、快速测定，满足大规模检测的需求，为烟草有机酸研究与烟叶品质评价提供了新的方法支撑。

## Abstract (en)

Organic acids are important components of dry tobacco and significantly affect its sensory properties. Therefore， determining the organic-acid composition and content in tobacco is greatly significant for evaluating its quality. Organic acids in tobacco are classified according to volatility as either volatile， semi-volatile， or non-volatile， with semi-volatile and non-volatile organic acids typically methylated for identification purposes. However， volatile acids cannot be determined in this manner owing to their low molecular weights. Therefore， a comprehensive， efficient， and accurate method for rapidly and simultaneously determining volatile， semi-volatile， and non-volatile organic acids in tobacco needs to be established. Herein， an innovative gas-chromatography–mass-spectrometry-based （GC-MS-based） isopropyl-esterification method was developed for simultaneously determining 29 volatile， semi-volatile， and non-volatile organic acids in tobacco. The developed method provides esterified small-molecule acids of lower volatility than those obtained using classical methylation and avoids interference from byproducts obtained via the esterification reaction. Experimental conditions and GC parameters were optimized， which ultimately led to 10% sulfuric acid in isopropanol （containing 0.1 g/mL *p*-toluenesulfonic acid） as the esterification reagent， with the reaction performed for 3 h at 70 °C and 500 r/min. The mixture was then cooled to room temperature， 10 mL of deionized water and 2 mL of *n*-hexane were added， and the mixture was vortexed for 30 s （4×）， after which the supernatant was collected for analysis. The DB-FASTFAME （30 m×0.25 mm×0.25 μm） and DB-5MS （30 m×0.25 mm×0.25 μm） chromatographic columns were compared， with the former finally selected for GC-MS separation and quantitative detection. All method-validation experiments were subsequently performed under the established instrumental and derivatization conditions. The 29 organic acids exhibited good linearities within the detection range， with all correlation coefficients （*r* ^2^） exceeding 0.99. All recoveries at three spiked levels ranged from 81.8% to 118.7%. The intra-day repeatabilities （RSD， *n*=6） ranged from 0.5% to 8.1%， and the inter-day repeatabilities （RSD， *n*=6） ranged from 0.9% to 8.2%. The limits of detection （*S/N*=3） were 0.01-49.02 μg/g， and the limits of quantification （*S/N*=10） were 0.03-163.41 μg/g， respectively. Comparative experimental results revealed that the developed method is highly consistent with the classical methylation-based derivatization method for the detection of semi- and non-volatile organic acids in tobacco. Furthermore， the new method is able to simultaneously determine volatile organic acids that cannot be analyzed using the classical methylation method. Therefore， the developed method is more widely applicable for determining acids in tobacco. The newly developed method was used to determine three types of tobacco sample： flue-cured tobacco， cigar tobacco， and burley tobacco， with all 29 organic acids successfully detected. Cigar tobacco leaves were found to contain significantly more volatile organic acids than flue-cured tobacco， especially 3-methylvaleric acid； they also contain less semi-volatile organic acids and more non-volatile organic acids than flue-cured tobacco. The developed method is simple， accurate， and suitable for the large-scale simultaneous determination of volatile， semi-volatile， and non-volatile organic acids in tobacco； it also provides additional technical support for the study of organic acids in tobacco and evaluating tobacco quality.

烟草中的有机酸一般指烟草中除氨基酸外的多元酸、脂肪酸、芳香酸等^［1］^。按照挥发性，烟草中的有机酸又划分为以C10以下、低级脂肪酸为主的挥发性小分子有机酸，如2-甲基丁酸、异戊酸、3-甲基戊酸等^［2，3］^，以及以多元酸和脂肪酸为主的非挥发性、半挥发性有机酸，如草酸、苹果酸、柠檬酸，以及棕榈酸、硬脂酸和油酸等^［4，5］^。有机酸具有独特的香味和酸性，挥发性的小分子有机酸可以直接转移至烟气中，对烟草香气有直接贡献^［6］^；半挥发性和非挥发性有机酸可以与烟草中的碱性物质结合，起到调节烟气酸碱平衡的作用^［7］^，对卷烟感官品质具有重要影响^［8］^。因此，准确、快速测定烟草中的多种有机酸，对烟草品质评价具有重要意义。

目前已报道多种有机酸测定的分析技术。如，相分离萃取后进行离子色谱-串联质谱法分析^［9-12］^、涡旋提取后进行超高效液相色谱-串联质谱法分析^［4，13，14］^，这类方法无需进行衍生化处理，多用于测定样品中的半挥发性和非挥发性有机酸。气相色谱-质谱联用法（GC-MS），因其灵敏度高、分离效果好、快速方便，是最常用的有机酸分析技术^［15］^，但一般需衍生化前处理以降低有机酸的沸点，改善其色谱分离性能。常用的有机酸衍生化方式主要包括硅烷化^［16，17］^与甲酯化^［5，8］^，硅烷化衍生多涉及溶剂浓缩、皂化等步骤^［7，18］^，前处理过程较为繁琐，不适于大批量样品的检测。相比之下，甲酯化衍生因其试剂易得、操作简单、结果准确等特点，已成为测定有机酸的传统经典方法，被广泛应用于烟草^［19，20］^、食品^［21，22］^等不同行业，并已作为国家和行业标准方法进行推广应用^［23-25］^。但由于挥发性有机酸甲酯的沸点低，易与溶剂共流出而无法实现分离测定，因此甲酯化衍生的前处理方式并不适于挥发性有机酸的测定，仅能测定半挥发性、非挥发性有机酸^［7］^。而在目前的有机酸酯化衍生体系研究中，除甲酯化衍生外，其他更长碳链的酯化衍生方式鲜有报道。因此，亟需开发一种操作简便、结果准确、检测通量高的酯化衍生方法，实现烟草中挥发性、半挥发性及非挥发性有机酸的同时、准确、快速测定。

基于上述问题，本研究基于硫酸异丙醇衍生体系，通过优化衍生反应中的关键参数，包括对甲苯磺酸添加量、衍生温度、时间、萃取溶剂等，建立了一种可以同时测定烟草中29种挥发性、半挥发性及非挥发性有机酸的异丙酯化衍生-气相色谱-质谱分析方法。并将该方法成功应用于不同类型烟草样品的分析，为烟草中有机酸类的测定提供了准确、快速的方法支撑。

## 1 实验部分

### 1.1 仪器、试剂与材料

气相色谱-质谱联用仪（7890B-5977A，Agilent），具有选择离子监测（SIM）功能；毛细管柱（DB-FASTFAME，Agilent），规格为（30 m×0.25 mm×0.25 μm）；Agitator振荡孵育器（G7379A，Agilent）；分析天平（XP603S，Mettler Toledo）；涡旋振荡仪（Vortex-Genie 2，Scientific Industries）。

29种有机酸标准品（纯度均≥98%，百灵威科技有限公司）；反式-3-己烯酸、己二酸标准品（纯度≥98%，上海安谱实验科技股份有限公司）；正己烷、异丙醇（色谱纯，Sigma-Aldrich公司）；浓硫酸（分析纯，国药集团化学试剂有限公司）；去离子水（Millipore纯水机制备）。

### 1.2 标准工作曲线配制

内标溶液：准确称取50.0 mg反式-3-己烯酸和1.0 g己二酸（精确至0.1 mg），用异丙醇定容至100 mL。

混合标准储备液：准确称取适量各有机酸标准物质，用异丙醇定容至刻度线，混匀，得到0.8 μg/mL~50.0 mg/mL的有机酸混合标准储备液。

标准工作溶液：分别移取0.01、0.02、0.05、0.10、0.20、0.50、1.00、2.00、5.00 mL混合标准储备液置于10 mL容量瓶中，用异丙醇定容至刻度线，混匀。分别移取1.0 mL上述溶液，置于20 mL顶空瓶中，依次加入100 μL内标溶液，2 mL 10%硫酸异丙醇溶液（含0.1 g/mL对甲苯磺酸），涡旋2 min，在70 ℃条件下振荡加热3 h，转速500 r/min。静置至室温后，加入10 mL去离子水、2 mL正己烷，涡旋振荡4次、每次30 s，静置分层，取上层清液加入2 mL色谱瓶中，得到标准工作溶液。

### 1.3 样品前处理

烟草样品在40 ℃下烘干10 h，磨粉后过孔径0.178 mm的筛网。准确称取0.1 g（精确至0.1 mg）样品粉末于 20 mL具盖顶空玻璃瓶中，加入100 μL内标溶液，2 mL 10%硫酸异丙醇溶液（含0.1 g/mL对甲苯磺酸），涡旋2 min，在70 ℃条件下中振荡加热3 h，转速500 r/min。静置至室温后，加入10 mL去离子水、2 mL正己烷，涡旋振荡4次、每次30 s，静置分层，取上层清液加入2 mL色谱瓶中，进行GC-MS上机分析。

### 1.4 仪器条件

#### 1.4.1 色谱条件

DB-FASTFAME色谱柱（30 m×0.25 mm×0.25 μm），进样口温度280 ℃；采用分流进样模式，分流比5∶1，进样量1 μL；载气为氦气（He，纯度>99.9%），采用恒流模式，流量为1.2 mL/min；程序升温条件设置如下：初始温度40 ℃，保持2 min，以5 ℃/min的速率升温至240 ℃，保持15 min。

#### 1.4.2 质谱条件

传输线温度280 ℃；电离方式为电子轰击源（EI），电离能量70 eV；离子源温度230 ℃；四极杆温度150 ℃；扫描模式为选择离子监测与全扫描，全扫描范围为*m/z* 33～550；溶剂延迟设置为2.7 min。29种有机酸异丙酯保留时间及离子信息见表1。

**表 1:** 29种有机酸异丙酯的保留时间、定量及定性离子

No.	Compound	Retention time/min	Quantitative ion （*m/z*）	Qualitative ion （*m/z*）	IS
1	formic acid isopropyl ester	3.03	73.0	45.0	IS1
2	acetic acid isopropyl ester	3.60	87.0	61.0	IS1
3	propanoic acid isopropyl ester	4.73	75.0	57.0	IS1
4	isobutyric acid isopropyl ester	5.00	89.0	71.0	IS1
5	butyric acid isopropyl ester	6.32	89.0	71.0	IS1
6	2-methylbutyric acid isopropyl ester	6.81	85.0	57.0	IS1
7	isovaleric acid isopropyl ester	7.22	103.0	85.0	IS1
8	valeric acid isopropyl ester	8.59	103.0	85.0	IS1
9	3-methylvaleric acid isopropyl ester	9.78	117.0	99.0	IS1
10	4-methylvaleric acid isopropyl ester	10.01	117.0	99.0	IS1
11	hexanoic acid isopropyl ester	10.97	117.0	99.0	IS1
12	heptanoic acid isopropyl ester	13.36	131.0	113.0	IS1
13	octanoic acid isopropyl ester	15.68	145.0	127.0	IS1
14	oxalic acid isopropyl ester	16.66	59.0	43.0	IS2
15	nonanoic acid isopropyl ester	17.89	159.0	141.0	IS1
16	propandioic acid isopropyl ester	18.33	105.0	129.0	IS2
17	benzoic acid isopropyl ester	19.67	123.0	105.0	IS1
18	decanoic acid isopropyl ester	19.99	173.0	155.0	IS1
19	phenylacetic acid isopropyl ester	21.99	178.0	91.0	IS1
20	dodecanoic acid isopropyl ester	23.87	183.0	200.0	IS2
21	malic acid isopropyl ester	26.14	131.0	89.0	IS2
22	myristic acid isopropyl ester	27.35	211.1	228.0	IS2
23	palmitic acid isopropyl ester	30.52	239.0	256.0	IS2
24	stearic acid isopropyl ester	33.43	267.0	284.0	IS2
25	oleic acid isopropyl ester	33.68	265.0	324.0	IS2
26	citric acid isopropyl ester	33.77	171.0	189.0	IS2
27	linoleic acid isopropyl ester	34.45	279.0	263.0	IS2
28	*α*-linolenic acid isopropyl ester	34.80	175.0	277.2	IS2
29	*γ*-linolenic acid isopropyl ester	35.29	261.0	277.0	IS2
30	*trans*-3-hexenoic acid isopropyl ester （IS1）	11.82	114.0	69.0	
31	adipic acid isopropyl ester （IS2）	24.99	129.0	171.0	

## 2 结果与讨论

### 2.1 实验条件考察

#### 2.1.1 酯化试剂的选择

有机酸酯化衍生是在强酸环境下，有机酸与醇类试剂反应生成相应的有机酸酯，可降低待测物沸点、改善目标化合物色谱分离性能，满足有机酸的检测需求。酯化反应的碳原子数越多，其生成的酯化产物沸点越高，越有利于小分子有机酸的检测^［26］^。实验发现甲醇、乙醇、丙醇等醇类试剂在强酸催化下，会产生甲酸甲酯、乙酸乙酯、丙酸正丙酯等副产物，干扰小分子挥发性有机酸的检测。本研究发现，异丙醇也可用作酯化反应的醇类试剂，反应生成有机酸异丙酯，且其在强酸催化下的副产物是异丙醚，不干扰丙酸的测定，因此本方法最终采用异丙醇的强酸溶液作为酯化试剂进行研究。

#### 2.1.2 色谱柱的优化

目前在有机酸甲酯化衍生的应用中，研究者多用DB-5MS或类似填料色谱柱^［5，7，30］^。本研究优先考察了DB-5色谱柱（30 m×0.25 mm×0.25 μm）对有机酸异丙酯类的分离效果，发现甲酸异丙酯在DB-5色谱柱中与异丙醇共流出，且无法通过选择离子监测的方式对其进行有效分离、定量。DB-FASTFAME（30 m×0.25 mm×0.25 μm）及DB-FATWAX（30 m×0.25 mm×0.25 μm）色谱柱均是为快速分离脂肪酸甲酯而设计的专用色谱柱，实验考察了两种色谱柱对29种有机酸异丙酯的分离效果。对两种色谱柱的升温程序分别进行优化后，结果发现DB-FASTFAME和DB-FATWAX色谱柱均能较好分离各有机酸异丙酯，而DB-FASTFAME色谱柱峰形略好，且异丙醇与前后目标化合物的分离效果也略好，因此选用DB-FASTFAME色谱柱用于后续研究。DB-FASTFAME柱对29种有机酸异丙酯的分离色谱图如图1所示。总离子流图中29种有机酸异丙酯均可在DB-FASTFAME色谱柱上实现良好分离。

**图1:**
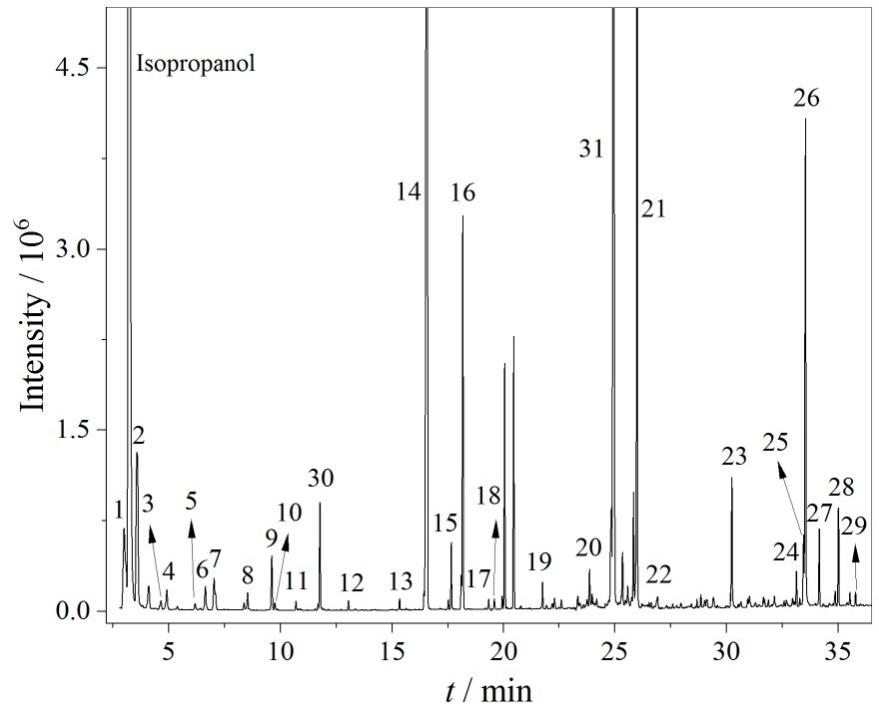
DB-FSATFAME色谱柱分离29种有机酸异丙酯的总离子流色谱图

#### 2.1.3 酯化衍生条件的优化

对甲苯磺酸是常见的有机强酸，文献报道适量添加对甲苯磺酸有助于提高酯化反应效率，缩短反应时间^［27］^。文章以10%硫酸异丙醇溶液的酯化体系为基础，考察了不同对甲苯磺酸添加量对酯化衍生的影响。对甲苯磺酸添加量分别为0、0.1、0.2、0.3、0.4 g/mL，反应时间均为3 h，反应温度均为70 ℃。将不同对甲苯磺酸添加量时各物质响应值除以对甲苯磺酸含量为0时各物质响应值，对数据进行归一化处理，实验结果如图2a所示。大多数挥发性、半挥发性和非挥发性有机酸异丙酯的响应随着对甲苯磺酸的加入而升高，且当含量为0.1 g/mL时响应最高，再增加对甲苯磺酸，挥发性有机酸异丙酯的响应无明显变化。而苹果酸、棕榈酸和硬脂酸等半挥发性及非挥发性有机酸异丙酯，当对甲苯磺酸含量升至0.2 g/mL或以上时，其响应明显逐步下降，表明更高的酸性会导致其有机酸异丙酯的分解。因此，最终采用含0.1 g/mL对甲苯磺酸的10%硫酸异丙醇作为酯化试剂。

**图2:**
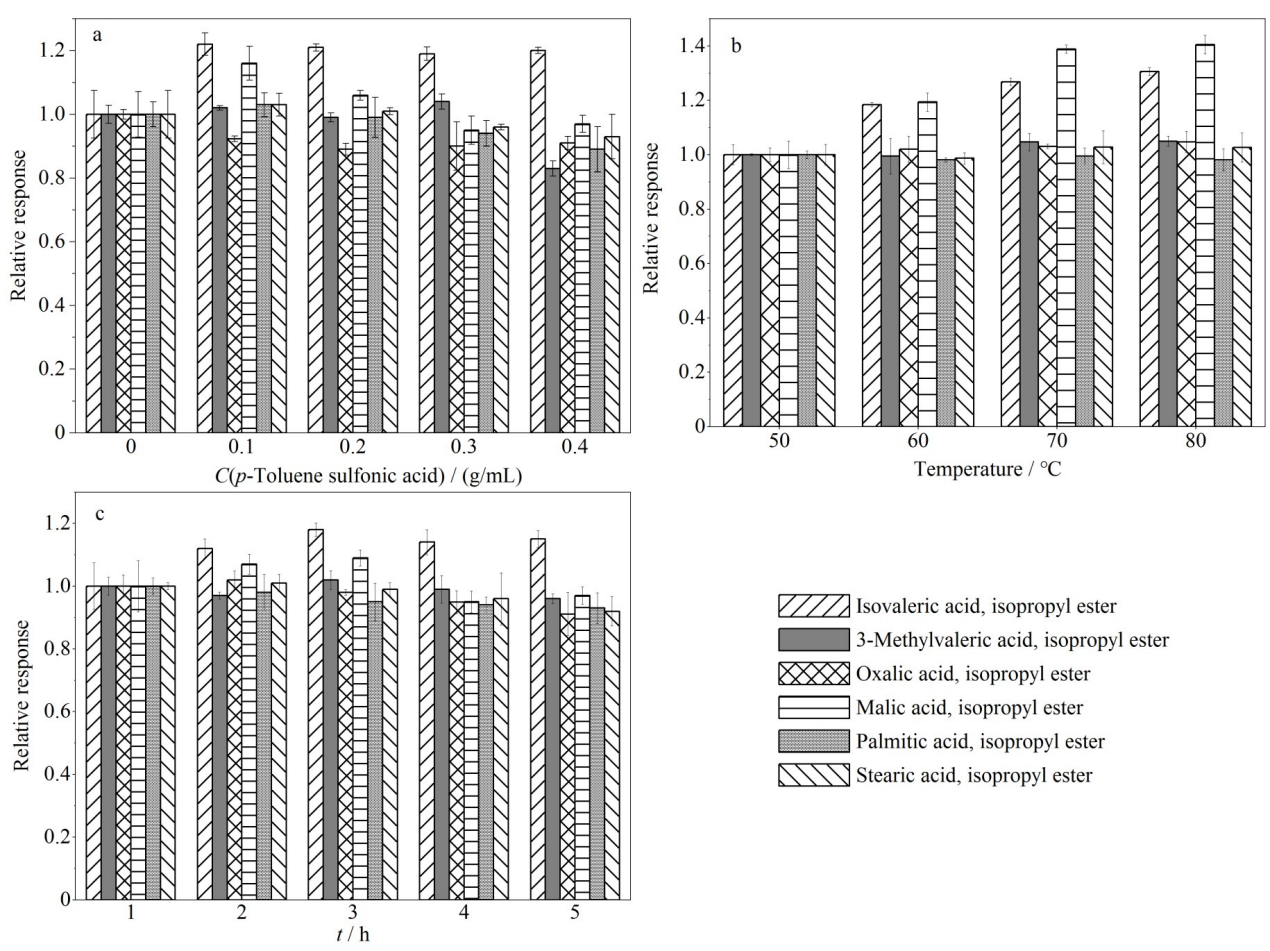
不同酯化衍生化条件对响应强度的影响（*n*=3）

考察了反应温度对酯化衍生的影响，衍生反应温度分别为50、60、70、80 ℃，时间均为3 h。将不同反应温度时各物质响应值除以50 ℃各物质响应值，对数据进行归一化处理，实验结果如图2b所示。结果表明，各有机酸异丙酯响应随着反应温度升高而逐步升高，绝大部分有机酸异丙酯在70 ℃时响应达到最高，且进一步升高温度各化合物响应提升并不明显，因此最终的酯化反应温度确定为70 ℃。

考察了反应时间对酯化衍生的影响，衍生反应时间分别为1、2、3、4、5 h，温度均为70 ℃。将不同反应时间时各物质响应值除以加热时间为1 h时各物质响应值，对数据进行归一化处理，实验结果如图2c所示。结果表明，对挥发性有机酸异丙酯来说，延长反应时间可提高其响应，而当反应时间长至3 h或更长，部分半挥发性、非挥发性有机酸异丙酯的响应出现下降，因此最终的酯化反应时间确定为3 h。

#### 2.1.4 萃取条件的优化

正己烷^［23，24］^、二氯甲烷^［25］^是有机酸酯化后的常用萃取溶剂，因此，文章考察了正己烷、二氯甲烷对有机酸异丙酯的萃取效果。实验固定加入2 mL萃取溶剂与10 mL去离子水，结果表明，在二氯甲烷萃取体系中，甲酸异丙酯无法在初始阶段与溶剂实现分离，且在后续有机溶剂与水层的液液萃取中，正己烷处于液面上层，更便于实验操作，因此本方法选用正己烷作为萃取溶剂。

在本衍生体系中，异丙醇的出峰位置在甲酸异丙酯和乙酸异丙酯中间，如萃取液含有较多异丙醇，会对目标物的色谱分离产生较大影响。因此在酯化反应完成后加入适量去离子水，一是快速终止酯化反应，二是降低萃取溶剂对异丙醇的萃取效率。研究分别考察了添加2、5、10 mL去离子水对检测结果的影响，萃取溶剂均为2 mL正己烷，结果如图3所示。随着去离子水体积的增加，异丙醇响应明显降低，且异丙醇与相邻的甲酸异丙酯、乙酸异丙酯的分离度明显改善，当去离子水添加量升至10 mL时，异丙醇与其前后的甲酸异丙酯、乙酸异丙酯实现完全基线分离，具备良好的分离效果，因此最终确定的去离子水添加量为10 mL。

**图3:**
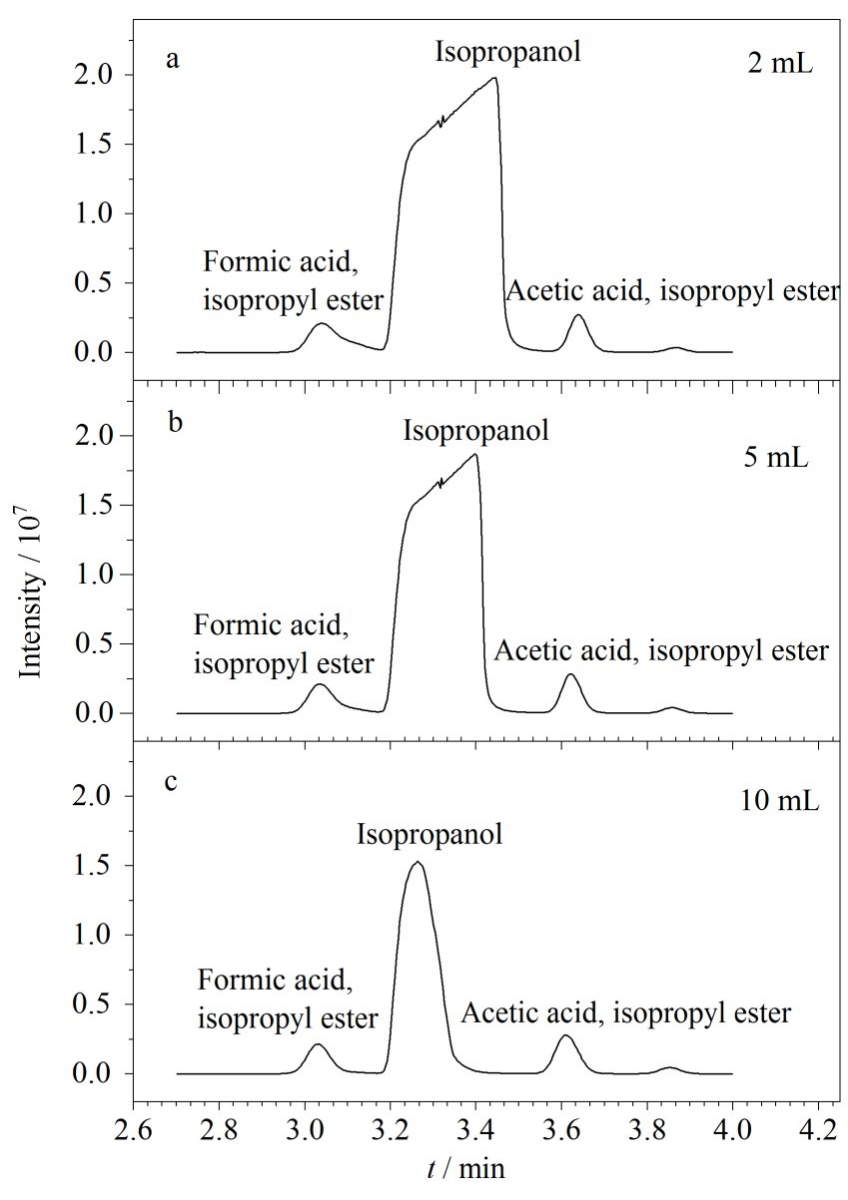
加水量对响应强度的影响

### 2.2 方法学考察

#### 2.2.1 标准曲线、检出限及定量限

在优化条件下，对1.4节中的29种有机酸混合标准工作溶液进行测定，以不同有机酸衍生产物与内标物峰面积的比值（*y*）为纵坐标，其对应的有机酸含量（*x*， μg/g）为横坐标，绘制标准工作曲线。结果表明，29种有机酸在表2所示的各自含量范围内线性关系良好，相关系数（*r*^2^） 均大于0.99。分别以色谱峰*S/N*=3和*S/N*=10对应的目标物含量确定方法的检出限（LOD）和定量限（LOQ），分别为0.01~49.02 μg/g和0.03~163.41 μg/g，结果如表2所示。

**表 2:** 29种有机酸的线性方程、相关系数、线性范围、检出限及定量限

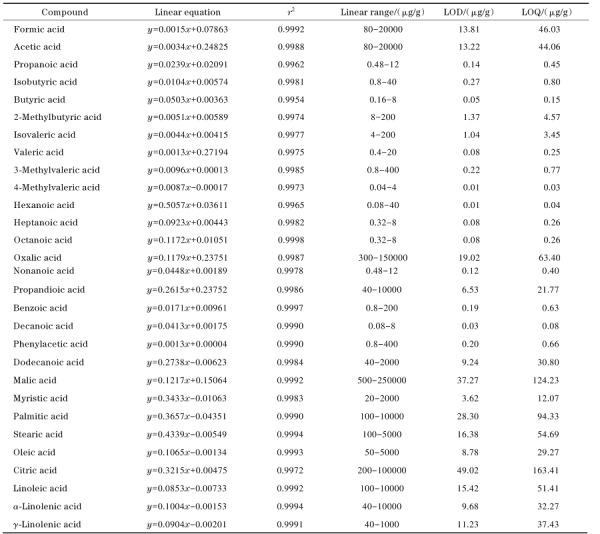

*y*： peak area ratio of analyte to internal standard； *x*： content， μg/g.

#### 2.2.2 重复性和回收率

以烤烟、雪茄烟和白肋烟质量比1∶1∶1混合作为研究基质，按照1.3节所述处理样品，每2 h进样一次，连续进样6次，考察方法的日内重复性；同时，每隔24 h进样一次，连续进样6次，考察方法的日间重复性。根据研究基质中各有机酸实际浓度，添加低、中、高3个加标水平的有机酸混合标准溶液进行加标回收率考察。结果表明，29种有机酸的加标回收率均介于80%~120%，满足定量检测要求（见表3）。29种有机酸检测结果的日内、日间重复性均在10%以内，且大多在5%以内，满足方法学考察的要求，适用于烟草中有机酸的含量测定。

**表 3:** 29种有机酸的加标回收率与日内、日间重复性 （*n*=6）

Compound	Low		Medium		High		RSDs/%	
	Added/（μg/g）	Recovery/%	Added/（μg/g）	Recovery/%	Added/（μg/g）	Recovery/%	Intra-day	Inter-day
Formic acid	1000.0	91.4	2000.0	111.3	6000.0	88.2	1.7	2.1
Acetic acid	1000.0	117.4	2000.0	108.2	6000.0	92.7	2.4	2.4
Propanoic acid	1.2	95.5	2.4	83.5	7.2	107.1	4.0	3.5
Isobutyric acid	4.0	89.7	8.0	108.3	24.0	93.5	8.1	7.9
Butyric acid	0.4	99.5	0.8	112.6	2.4	94.7	7.8	8.2
2-Methylbutyric acid	20.0	98.0	40.0	118.7	120.0	100.2	3.9	3.6
Isovaleric acid	10.0	96.9	20.0	107.6	60.0	83.7	2.9	3.0
Valeric acid	1.0	89.1	2.0	84.5	6.0	84.9	5.4	5.6
3-Methylvaleric acid	40.0	100.1	80.0	114.3	160.0	95.6	5.9	4.9
4-Methylvaleric acid	0.2	90.7	0.4	102.5	1.2	113.2	3.1	4.3
Hexanoic acid	2.0	102.8	4.0	88.4	12.0	92.4	7.1	8.1
Heptanoic acid	0.4	90.5	0.8	89.2	2.4	90.1	4.9	5.3
Octanoic acid	0.4	81.8	0.8	100.0	2.4	93.4	2.4	3.6
Oxalic acid	7500.0	97.9	15000.0	111.3	45000.0	115.1	0.5	1.1
Nonanoic acid	1.2	100.2	2.4	104.2	7.2	101.7	7.2	7.1
Propandioic acid	500.0	89.0	1000.0	93.2	3000.0	98.7	0.6	1.0
Benzoic acid	10.0	86.0	20.0	98.4	60.0	112.7	4.0	3.7
Decanoic acid	0.8	98.6	1.6	94.2	4.8	97.3	3.8	6.1
Phenylacetic acid	20.0	97.4	40.0	96.3	120.0	82.6	3.0	2.1
Dodecanoic acid	100.0	96.3	200.0	98.8	600.0	106.7	8.1	7.6
Malic acid	12500.0	110.5	25000.0	103.1	75000.0	96.2	0.9	0.8
Myristic acid	100.0	112.1	200.0	107.9	600.0	103.2	4.2	5.8
Palmitic acid	500.0	83.9	1000.0	110.2	3000.0	108.7	0.8	1.0
Stearic acid	250.0	93.2	500.0	99.7	1500.0	116.4	1.8	3.2
Oleic acid	250.0	99.5	500.0	101.5	1500.0	104.8	4.2	6.2
Citric acid	5000.0	103.0	10000.0	109.2	30000.0	115.6	0.8	1.0
Linoleic acid	500.0	88.1	1000.0	104.7	3000.0	116.3	1.7	2.1
*α*-Linolenic acid	500.0	110.2	1000.0	85.8	3000.0	116.7	2.6	1.9
*γ*-Linolenic acid	50.0	103.9	100.0	109.2	300.0	96.1	1.9	2.1

#### 2.2.3 与甲酯化衍生法的比对

甲酯化衍生-气相色谱法作为测定半挥发性及非挥发性有机酸的经典方法^［28］^，在烟草行业具有广泛的应用，也是研究烟草中半挥发性及非挥发性有机酸含量特性的首选检测方法^［29］^。采用甲酯化衍生法^［25］^与本文所建立异丙酯衍生化方法对烟草样品中的半挥发性及非挥发性有机酸进行定量检测，甲酯化衍生-气相色谱法未检测到含量较低的肉豆蔻酸、月桂酸和*γ*-亚麻酸，其余有机酸检测结果见图4。由图4可知，在测定半挥发性及非挥发性有机酸方面，本方法与经典的甲酯化衍生法的测定结果具有高度一致性。除此之外，对于甲酯化衍生法无法检测的挥发性有机酸，本方法可以实现其与半挥发性及非挥发性有机酸的同时定量检测，比甲酯化衍生法具备更广泛的检测范围，可以同时测定更多种类的有机酸。

**图4:**
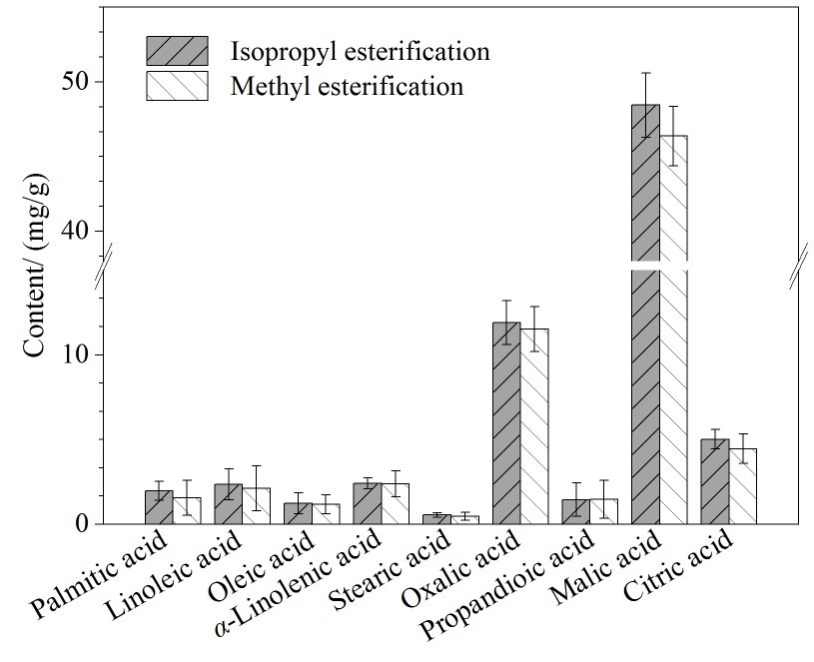
异丙酯化与甲酯化检测结果的比较（*n*=3）

### 2.3 实际样品的检测结果

采用新方法对烤烟、雪茄烟、白肋烟3种不同类型烟叶样品中的有机酸进行了检测，其具体检测结果如表4所示。由表中数据可以看出，烤烟中甲酸、乙酸、2-甲基丁酸等的含量高于雪茄烟和白肋烟，雪茄烟中3-甲基戊酸、苯乙酸的含量显著高于白肋烟和烤烟；雪茄烟、白肋烟中草酸、月桂酸、肉豆蔻酸、柠檬酸等有机酸含量大多显著高于烤烟，而棕榈酸、硬脂酸、油酸、亚油酸、*α*-亚麻酸等的含量显著低于烤烟，这与朱玲等^［30］^采用甲酯化衍生法对雪茄烟与烤烟中半挥发性及非挥发性有机酸含量的检测结果基本一致。

**表 4:** 不同类型烟叶样品中29种有机酸的检测结果

Compound	Contents/（μg/g）		
	Flue-cured tobacco	Cigar tobacco	Burley tobacco
Formic acid	4040.12	915.35	833.92
Acetic acid	2990.62	2840.36	1842.75
Propanoic acid	1.79	2.17	4.51
Isobutyric acid	4.06	10.89	15.34
Butyric acid	1.27	0.55	0.66
2-Methylbutyric acid	77.21	29.82	46.82
Isovaleric acid	39.82	15.73	45.12
Valeric acid	1.13	3.44	3.56
3-Methylvaleric acid	1.65	332.62	2.43
4-Methylvaleric acid	0.21	0.34	0.19
Hexanoic acid	2.25	5.29	2.61
Heptanoic acid	1.05	0.72	1.08
Octanoic acid	1.95	1.42	0.78
Oxalic acid	11912.24	43740.71	27642.30
Nonanoic acid	2.28	3.96	2.19
Propandioic acid	1455.76	3207.07	1095.43
Benzoic acid	21.40	21.55	13.73
Decanoic acid	0.87	0.56	1.83
Phenylacetic acid	13.31	111.97	52.60
Dodecanoic acid	230.27	542.73	373.21
Malic acid	48292.83	33982.35	79813.62
Myristic acid	122.22	279.94	314.14
Palmitic acid	1971.21	1046.90	895.36
Stearic acid	452.12	298.69	282.36
Oleic acid	822.23	422.12	70.78
Citric acid	5026.46	38582.12	46874.52
Linoleic acid	2354.47	838.69	1682.12
*α*-Linolenic acid	2422.73	330.71	720.71
*γ*-Linolenic acid	134.91	133.74	124.55

## 3 结论

本研究开发了一种异丙酯化衍生-气相色谱-质谱法同时测定烟草中29种有机酸的高通量方法。该方法基于10%硫酸异丙醇溶液（含0.1 g/mL对甲苯磺酸）的异丙酯化衍生体系，具有良好的反应效率和分离效果，操作简单，灵敏度高，稳定性强，适用于不同类型烟草样品中挥发性、半挥发性及非挥发性有机酸的同时快速定量检测，为烟草中有机酸的研究提供了全新的方法手段，为烟草的品质评价提供了新的技术支撑。
